# Molecular features of lung adenocarcinoma in young patients

**DOI:** 10.1186/s12885-019-5978-5

**Published:** 2019-08-06

**Authors:** Zhen Chen, Xiao Teng, Jing Zhang, Ke Huang, Qian Shen, He Cao, Huisong Luo, Yanting Yuan, Xiaodong Teng

**Affiliations:** 1 Department of Pathology, the First Affiliated Hospital, College of Medicine, Zhejiang University, Hangzhou, 310000 China; 2 Department of Thoracic Surgery, the First Affiliated Hospital, College of Medicine, Zhejiang University, Hangzhou, 310000 China; 3 Department of Respiratory Diseases, the First Affiliated Hospital, College of Medicine, Zhejiang University, Hangzhou, 310000 China; 4 Singlera Genomics Inc., Shanghai, 200000 China

**Keywords:** Lung adenocarcinoma, Young age, Next-generation sequencing, Molecular features

## Abstract

**Background:**

Lung cancer in young patients is rare and has unique clinicopathological features. However, the molecular features of lung cancer in these patients are unclear. In this study, we aimed to describe the molecular features and outcomes of lung adenocarcinoma in patients aged ≤35 years.

**Methods:**

A total of 89 patients aged ≤35 years with pathologically diagnosed lung adenocarcinoma were retrospectively evaluated. Mutations in 59 cancer-associated genes and fusions of *ALK* and *ROS1* were analyzed to understand the molecular features of young patients with lung adenocarcinoma. The clinicopathological characteristics and prognosis of each patient were reviewed.

**Results:**

Of the 89 young patients, 25 (28.1%) were male, 9 (10.1%) were smokers, and the median age was 32 years (range, 18–35 years). The authors analyzed 59 genes and a total of 6 mutations and 2 fusion genes were detected. These genes were distributed among 60 patients, 12 of which had two or more mutations. *ERBB2* mutations were most common (24.7%), followed by *EGFR* mutation (21.3%), *ALK* fusion (16.9%), *TP53* mutation (9.0%), *BRAF* mutation (3.4%), *PIK3CA* mutation (1.1%), *CTNNB1* mutation (1.1%), and *ROS1* fusion (1.1%). *EGFR*, *ERBB2*, and *TP53* mutations, gene abnormalities, and *ALK* fusions all had significant correlations with histopathological differentiation (*P* < 0.01). *ALK* fusions and *EGFR* mutations conferred a significantly worse prognosis than did *ERBB2* mutations and tumors that contained no mutations or fusions (*P* < 0.01).

**Conclusions:**

The molecular features of lung adenocarcinoma in young patients are different from those of common adenocarcinoma, and the main driver genes are closely correlated with tumor differentiation and prognosis.

**Electronic supplementary material:**

The online version of this article (10.1186/s12885-019-5978-5) contains supplementary material, which is available to authorized users.

## Background

Lung cancer, which is a common geriatric disease, is one of the most lethal cancers in the world [1, 2], with an age of onset of approximately 60 years [2]. Lung cancer in young patients is rare; less than 3.5% of patients are younger than 45 years old [2]. It has been confirmed that young patients with lung cancer have different clinicopathological features than elderly patients, and female sex and adenocarcinoma predominance are consistent features [3, 4]. However, only a few studies have investigated the molecular features of lung cancer in young patients, and most have focused on the mutational frequency of several specific driver events involved in lung cancer. Previous studies have shown that young patients with lung cancer have a lower incidence of *epidermal growth factor receptor (EGFR)* mutations and a higher incidence of *anaplastic lymphoma kinase (ALK)* fusions [5–11], but not all studies have reported the same results [12, 13].

In this study, data from 89 patients with lung adenocarcinoma (LUAD) aged 35 years or younger were used to determine the mutation status of 59 cancer-related genes by next-generation sequencing (NGS) technology, and fusions of *ALK* and *c-ros oncogene 1 (ROS1)* were identified, which provides further insight into the molecular features of young patients with LUAD.

## Methods

### Patients and data collection

We retrospectively collected data from 89 patients aged 35 or younger with pathologically diagnosed LUAD between June 2014 and September 2016 at the First Affiliated Hospital, College of Medicine, Zhejiang University. All of the patients were treatment naïve; no radiotherapy, chemotherapy, or targeted therapy was administered before specimen collection. The histological type and differentiation determinations were made by two pathologists according to the 2015 WHO classification of lung neoplasms [14], and disease stage was determined based on the tumor-node-metastasis (TNM) classification of the Union for International Cancer Control (UICC) [15]. This study was approved by the Ethics Committee of the First Affiliated Hospital College of Medicine, Zhejiang University. All participants provided written informed consent.

### Sample preparation

Formalin-fixed and paraffin-embedded (FFPE) specimens with a high proportion of tumor cells are important for accurate analyses. Pathological quality control was performed before sample detection. Samples with a tumor cell proportion lower than 20% were removed from the study. Macro-dissection of tumors to enrich the tumor cell content was performed before FFPE DNA/RNA extraction according to the H&E staining results.

### DNA/RNA extraction

FFPE DNA was isolated using the QIAamp DNA FFPE Tissue Kit (Qiagen, Cat No./ID: 56404, Germany) according to the manufacturer’s instructions. FFPE DNA quality was evaluated by 1% agarose gel electrophoresis, and any FFPE DNA that was severely degraded was removed. DNA quantification was conducted with a Qubit 3.0 fluorometer and a Qubit dsDNA HS assay kit (Life Technologies, USA). FFPE RNA extraction was performed according to the manufacturer’s protocol (AmoyDx, Xiamen, China).

### NGS analysis

An amplicon-based targeted NGS assay was used for library preparation with an OncoAim™ Tumor Mutation and PharmGx Detection Kit from Singlera Genomics (covering > 6000 hotspot mutations in 59 common cancer-associated genes, Additional file 1: Table S1). Experiments were performed according to the kit instructions. Twenty nanograms of DNA was used for library preparation as recommended by the kit instructions. The library product was sequenced using 75-bp paired-end runs on the Illumina MiSeq after quantification using a KAPA Library Quantification Kit (Kapa, KK4824).

Sequencing data were processed by the manufacturer’s supplied bioinformatics software. The minimum depth and mutation frequency of high-quality single nucleotide polymorphisms (SNPs) and indels (≤50 bp) were set to ≥100x and ≥ 5%, respectively.

### *ROS1* and *ALK* fusion analysis

*ROS1* fusions were detected using a reverse transcription-polymerase chain reaction (RT-PCR) assay (AmoyDx, Xiamen, China). The tissue was treated as for DNA extraction, and RNA extraction was performed according to the manufacturer’s protocol (AmoyDx, Xiamen, China) and as described by Cai et al [16]. *ALK* fusions were detected by automated immunohistochemistry (IHC), which was performed at Ventana Medical Systems (Tucson, AZ, USA) on 3-μm-thick FFPE sections with the D5F3 rabbit anti-human monoclonal antibody (Cell Signaling Technologies Ventana-Roche, Tucson, AZ). We used binary classification to evaluate the IHC results obtained by two pathologists. A positive result was defined as strong granular staining of the tumor cell cytoplasm.

### Statistical analysis

The patients were followed until November 2018 or the date of their death. Overall survival (OS) was defined as the time from the date of diagnosis to the date of death or last visit. The Kaplan-Meier method with a log-rank test and Cox regression analysis were used for survival analyses. The correlation of different groups with clinicopathological characteristics was studied via chi-square test or Fisher’s test. A *P* value < 0.05 was considered statistically significant. Statistical analysis was performed using SPSS 17.0 software (Chicago, USA).

## Results

### Demographic characteristics of young patients with LUAD

Among the 89 patients, 25 were male, and 64 were female; the ratio of males to females was 1:2.56; and patients were aged 18–35 years, with a median age of 32 years. Nine patients smoked, and the remaining 80 had never smoked. In total, 54 cases with well differentiation, 13 cases with moderate differentiation, and 22 cases with poor differentiation. There were 63 patients with stage I disease, 5 with stage II disease, 6 with stage III disease, and 15 with stage IV disease. Details are shown in Table 1.

**Table 1:** Demographic characteristics of 89 young patients with LUAD

	N	%
Gender		
Male	25	28.1%
Female	64	71.9%
Differentiation		
Well	54	60.7%
Moderate	13	14.6%
Poor	22	24.7%
Clinical stage		
I	63	70.8%
II	5	5.6%
III	6	6.7%
IV	15	16.9%
Smoking status		
Smokers	9	10.1%
Never smoked	80	89.9%

### Gene abnormalities (GA) of young patients with LUAD

**Figure 1:**
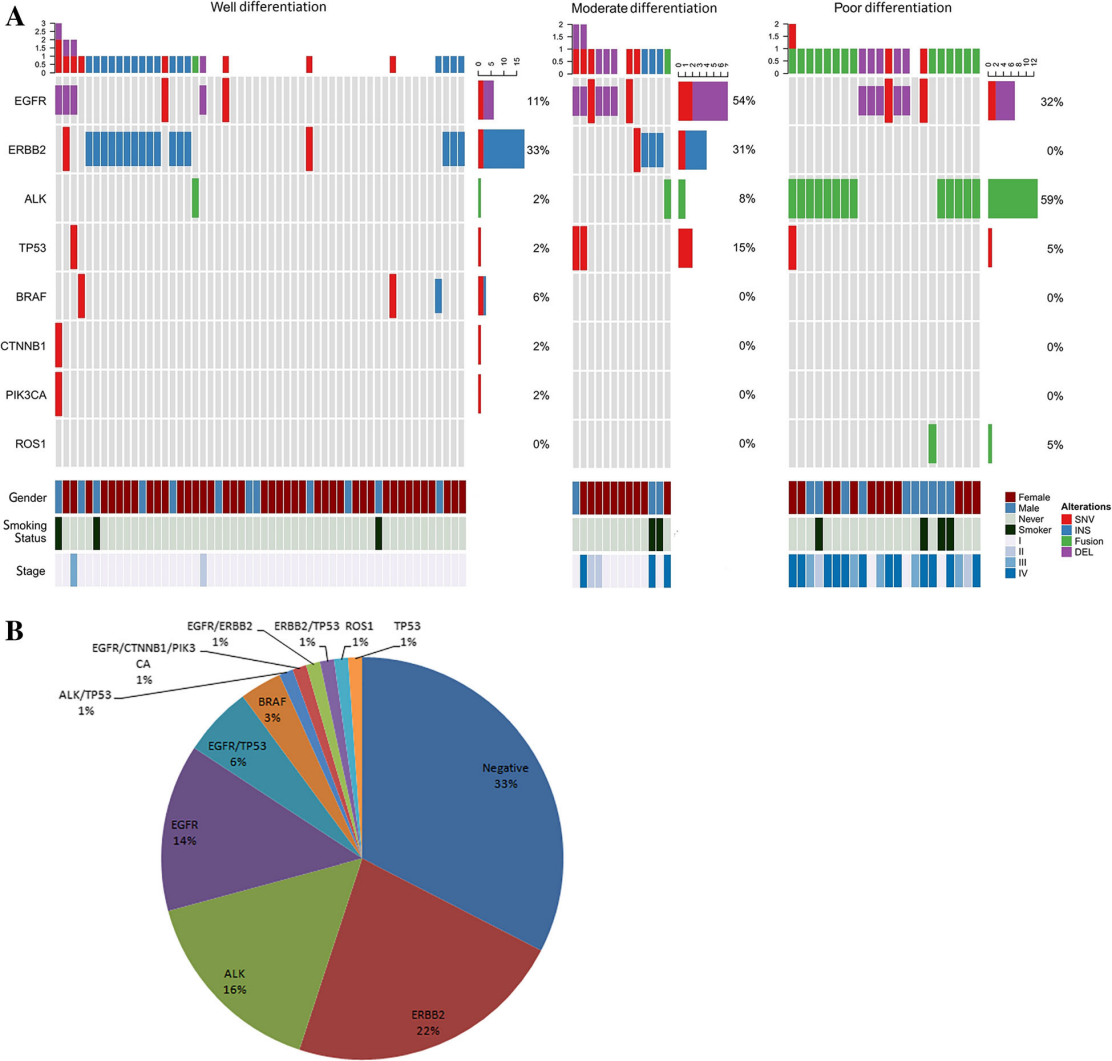
GA in 89 young patients with LUAD. A: Comutation plot of 89 LUAD s. B: Frequency of GA in young patients with LUAD

A total of 6 mutant genes and 2 fusion genes were detected and distributed among the 60 patients (Fig. 1), 12 of whom had two or more mutations. The most frequently affected genes were *ERBB2* and *EGFR*, with mutation rates of 24.7 and 21.3%, respectively, followed by *ALK* (16.9%), *TP53* (9.0%), *BRAF* (3.4%), *PIK3CA* (1.1%), *CTNNB1* (1.1%), and *ROS1* (1.1%) (Table 2).

**Table 2:** The frequency of gene mutations in 89 young patients with LUAD

Genes	Mutations	No.
*EGFR*	E746_A750delELREA	13
	L858R	1
	L858R/S768I	1
	L858R/E709A	1
	G719S/E709A	1
	L747S	1
	H773Y	1
*ERBB2*	A775_G776insYVMA	15
	G776 > VC	3
	P780_Y781insGSP	1
	S310F	1
	V659E	1
	L755P	1
*BRAF*	T599_V600insT	1
	L597R	1
	D594G	1
*PIK3CA*	E542K	1
*CTNNB1*	S33Y	1
*TP53*	D281H	2
	Q52*	1
	R175H	1
	C176F	1
	Y205C	1
	R280T	1
	R282W	1

### Correlation between GA and clinicopathological characteristics

GA were found more frequently in advanced-stage, poorly differentiated tumors than in early-stage, well-differentiated tumors (*P* < 0.01, Table 3). Significantly fewer *EGFR* mutations were identified in well-differentiated tumors than in moderately differentiated and poorly differentiated tumors (*P* < 0.01), with no statistically significant differences in gender, TNM stage, or smoking status. *ERBB2* mutations were significantly more common in well-differentiated tumors than in poorly differentiated tumors (*P* < 0.01), with no significant differences in gender or smoking status (Table 3). A total of 15 patients were found to be positive for *ALK* expression, and the *ALK*-positive rate was significantly higher in poorly differentiated tumors than in well-differentiated tumors (*P* < 0.01) and in advanced-stage disease than in early-stage disease (*P* < 0.01). The histomorphology was most cribriform arrangement or solid. No significant difference was found in gender or smoking status. Most *TP53* mutations co-occurred with other gene mutations and were significantly more common in well-differentiated tumors than in poorly differentiated tumors (*P* < 0.01). *CTNNB1* and *PIK3CA* mutations co-occurred with an *EGFR* mutation in one male patient, and *ROS1* fusion occurred in a 34-year-old male patient who had never smoked.

**Table 3:** Correlation between GA and clinicopathological characteristics in 89 young patients with LUAD

	ALL	GA	*P*	*EGFR*	*P*	*ERBB2*	*P*	*ALK*	*P*	*TP53*	*P*
Gender			0.280		0.846		0.922		0.620		1.000
Males	25	19(76.0%)		5(20.0%)		6(24.0%)		5(20.0%)		2(8.0%)	
Females	64	41(64.1%)		14(21.9%)		16(25.0%)		10(15.6%)		6(9.4%)	
Differentiation			0.000		0.001		0.000		0.000		0.001
Well	54	27(50.0%)		5(9.3%)		18(33.3%)		1(1.9%)		2(3.7%)	
Moderate	13	12(92.3%)		7(53.8%)		4(30.8%)		1(7.7%)		1(7.7%)	
Poor	22	21(95.5%)		7(31.8%)		0		13(59.1%)		5(22.7%)	
Stage			0.002		0.214		0.103		0.000		0.129
I-III	74	45(60.8%)		14(18.9%)		21(28.4%)		7(9.5%)		5(6.8%)	
IV	15	15(100%)		5(33.3%)		1(6.7%)		8(53.3%)		3(20.0%)	
Smoking status			0.261		1.000		0.684		0.173		0.590
Smokers	9	8(88.9%)		2(22.2%)		3(33.3%)		3(33.3%)		1(11.1%)	
Never	80	52(65.0%)		17(21.3%)		19(23.8%)		12(15.0%)		7(8.8%)	

### Survival analysis

**Figure 2:**
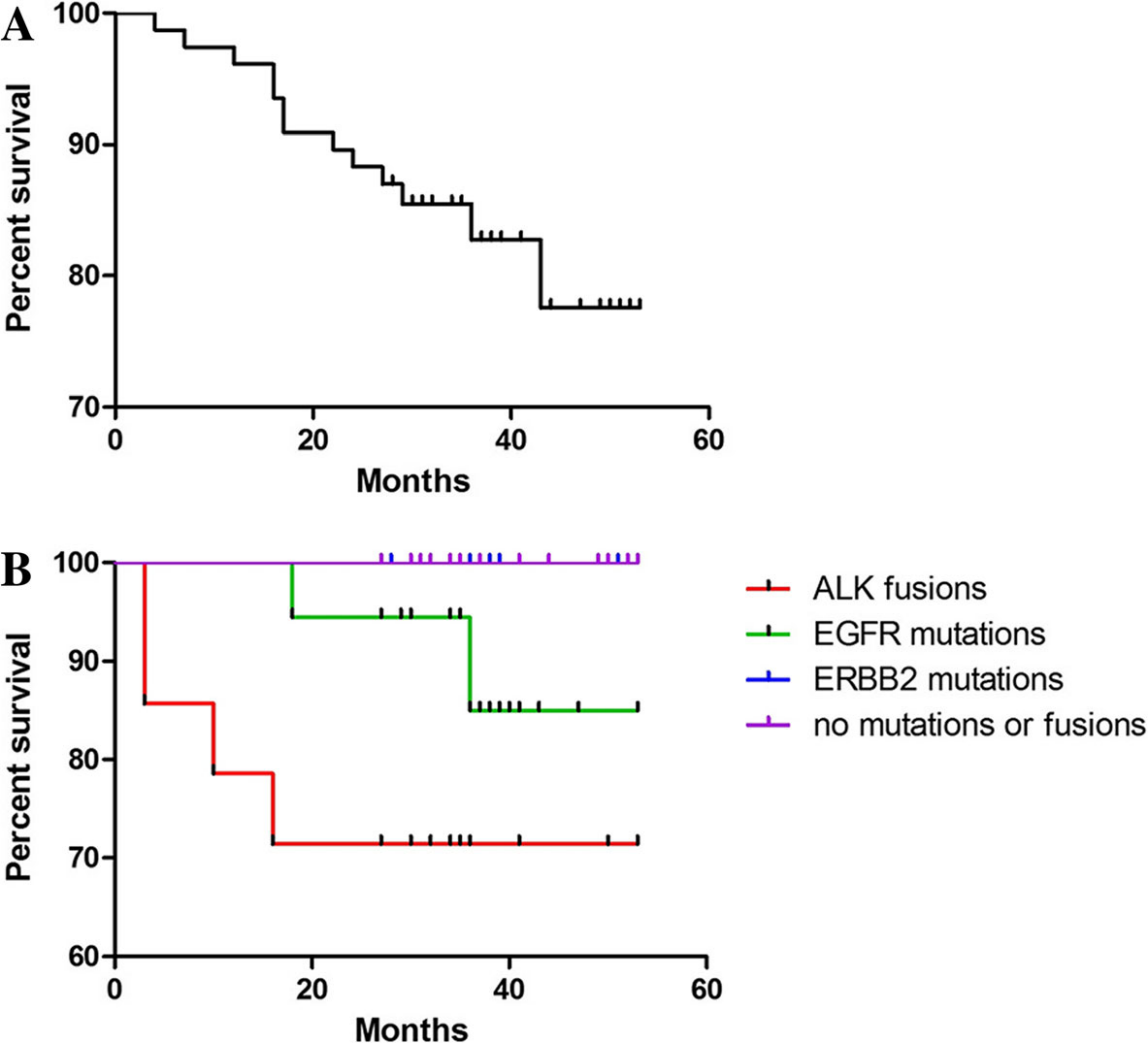
Survival curves for young patients with LUAD. A: OS curves for 83 patients. B: Survival by oncogenic drivers

Long-term follow-up data were available for 83 patients. The follow-up times ranged from 3 to 53 months (median time, 34 months). Twenty-three patients received tyrosine kinase inhibitor (TKI) treatment, of which 4 were treated with an EGFR-TKI, and 14 were treated with an ALK-TKI. Seven deaths occurred during the follow-up period. The median OS times were 53 months for patients with early-stage disease and 19 months for those with advanced disease. Patients with *ALK* fusions, poor differentiation or stage IV disease had a significantly worse prognosis (*P* < 0.01). *ALK* fusions and *EGFR* mutations conferred a significantly worse prognosis compared with *ERBB2* mutations and no mutations or fusions (*P* < 0.01, Fig. 2).

## Discussion

With improvements in awareness of physical examinations and the prevalence of improved computed tomography (CT) imaging with higher resolution, more young people are being diagnosed with lung cancer, and more early-stage lung cancers are being discovered. Similar to our previous study [17], most patients in the current cohort were female with early-stage cancer. It is generally believed that the occurrence of lung cancer is related to smoking, and smoking significantly increases the incidence of lung cancer [18]. However, in our cohort, the number of smokers was very small (9/89), suggesting only a loose relationship between the occurrence of LUAD and smoking in young patients.

To date, targeted drugs for driver genes have significantly improved the treatment of patients with lung cancer. Adrian et al compared the relationship between targetable genomic alterations and age in 2237 patients with lung cancer and found that young patients had higher frequencies of *EGFR*, *ALK*, *ROS1*, and *ERBB2* alterations [9]. Other studies have also reported a high frequency of *ALK* fusions in young patients with lung cancer [5, 7, 8, 10]. However, in East Asian populations, the frequency of *EGFR* mutations appears to be lower in younger patients than in elderly patients [7, 10, 11]. In the present study, we expanded the detection range to include mutations of 59 common cancer-associated genes and fusions of *ALK* and *ROS1*, and only a few classic lung cancer GA were observed. Eight GA were found to be distributed in 67.4% (60/89) of patients. Among them, *ERBB2* had the highest mutation frequency, at 24.7% (22/89), followed by *EGFR*, *ALK*, and *TP53*.

*ERBB2*, *EGFR*, and *TP53* mutations and *ALK* fusions were present at rates of approximately 2, 50, 50 and 7% in an unselected East Asian adenocarcinoma population [19–25]. In our cohort, *ERBB2* mutations and *ALK* fusions were significantly enriched, and the frequencies of *EGFR* and *TP53* mutations were significantly lower, similar to the results of previous studies. However, Ye et al reported that the frequencies of *TP53* mutations and *ALK* fusions in young patients with LUAD were 72.2 and 5.6% (2/36), respectively [12].

There were also significant differences in the survival of patients with *EGFR* mutations, *ERBB2* mutations, *ALK* fusions or no GA. Patients with *ERBB2* mutations or no GA had a significantly better OS than patients with *EGFR* mutations or *ALK* fusions.

Interestingly, *EGFR* and *ERBB2* mutations and *ALK* fusions also exhibited unique clinicopathological features in our cohort. Among the unselected adenocarcinoma patients, *EGFR* and *ERBB2* mutations and *ALK* fusions were frequent in nonsmoking female patients [20, 22, 24, 26]. However, in this study, *EGFR* and *TP53* mutations and *ALK* fusions were significantly more frequent in poorly differentiated cases than in well-differentiated cases, whereas *ERBB2* mutations were predominantly concentrated in well-differentiated cases. *EGFR* and *ERBB2* mutations and *ALK* fusions were not associated with patient gender or smoking status. The features of these driver genes in young patients with LUAD may be the reason for the low frequency of *ALK* fusions in the study by Ye et al (5.6%, 2/36) [12]. *ALK* fusions usually occur in patients with advanced adenocarcinoma, and patients with early-stage cancer composed the main population in the cohort studied by Ting and colleagues [12]. In this study, the overall positive rate of *ALK* fusion was 16%, and up to 59% of adenocarcinomas with poor differentiation exhibited an *ALK* fusion, consistent with previous reports [10]. As an important tumor suppressor gene, *TP53* mutation is widespread in a variety of tumors, which can lead to the inactivation of its protein [27]. Approximately 50% of lung cancers have *TP53* mutations [25]. Ye et al reported that the *TP53* mutation rate in young patients with LUAD was significantly higher than that in old patients (72.2% vs. 25.3%, *P* < 0.001) [12]. In our cohort, we did not find a higher frequency of *TP53* mutations (9%, 8/89), which were frequent in poorly differentiated cases. We are not sure whether the difference in the frequency of *TP53* mutations was due to the differences in tumor histopathological differentiation between the two study populations.

Multiple reports have shown that features of *ROS1* and *ALK* fusions are similar, and these fusions commonly occur in young patients who have never smoked and have a high grade of malignancy [28]. Adrian et al also found that young patients with lung cancer had a tendency toward *ROS1* fusion enrichment [9]. However, in this study, a high frequency of *ROS1* fusion was not found; only one instance was found in a male patient who had never smoked.

*BRAF* mutations, mostly V600E, occur in approximately 2% of non-small-cell lung cancer (NSCLC) cases [29]. In our cohort, *BRAF* mutations were found in 3 patients, none of which was V600E. There are no reports of an abnormal distribution of *BRAF* mutations in young lung cancer patients in the literature. *PIK3CA* and *CTNNB1* mutations are rare in LUAD [30, 31]. In our cohort, a male patient with *PIK3CA*, *CTNNB1* and *EGFR* comutations was found, and the patient was diagnosed with stage I disease. He remained stable after lobectomy until the last follow-up date.

## Conclusions

Overall, LUAD in young patients is a special type of lung cancer that exhibits molecular features that are different from common LUAD, and the main driver genes are closely correlated with tumor differentiation and prognosis. ERBB2 mutations are mainly distributed in well- and moderately differentiated tumors with good prognosis; EGFR mutations are mainly distributed in moderate- and poorly differentiated tumors, and the prognosis is relatively favorable; ALK fusions are mainly distributed in poorly differentiated tumors with a poor prognosis. We hope that this study will help guide clinicians in determining the appropriate therapy.

Although this study had the largest sample size of the studies on the molecular features of LUAD in young patients and was the first to describe the clinicopathological features of the main driver genes in this cohort, it has several shortcomings. (1) Although 59 cancer-associated genes were analyzed, many genes were not, and these genes may include abnormal genes that are specific to young patients with LUAD. (2) The NGS panel used in this study targeted only somatic DNA mutations, and fusion genes could not be detected. (3) The cohort was from a single center, and the sample size was small.

## Additional file

Additional file 1: Table S1. List of 59 cancer-associated genes. (DOC 35 kb)

## Data Availability

The data analyzed during the current study are available from the corresponding author on reasonable request.

## Abbreviations

*ALK*: anaplasticlymphoma kinase;
*BRAF*: B-Raf proto-oncogene;
*CTNNB1*: catenin beta 1
*EGFR*: epidermal growth factor receptor
*ERBB2*: erb-b2 receptor tyrosine kinase 2
FFPE: formalin-fixed and paraffin-embedded
GA: gene abnormalities
LUAD: lung adenocarcinoma
NGS: next-generation sequencing
OS: overall survival
*PIK3CA*: phosphatidylinositol-4,5-bisphosphate 3-kinase catalytic subunit alpha
*ROS1*: c-ros oncogene 1
TP53: tumor protein p53

## References

[1] Torre LA, Bray F, Siegel RL, Ferlay J, Lortet-Tieulent J, Jemal A (2015). Global cancer statistics, 2012. CA Cancer J Clin.

[2] Chen W, Zheng R, Baade PD, Zhang S, Zeng H, Bray F, Jemal A, Yu XQ, He J (2016). Cancer statistics in China, 2015. CA Cancer J Clin.

[3] Kuo CW, Chen YM, Chao JY, Tsai CM, Perng RP (2000). Non-small cell lung cancer in very young and very old patients. Chest.

[4] Maruyama R, Yoshino I, Yohena T, Uehara T, Kanematsu T, Kitajima M, Teruya T, Ichinose Y (2001). Lung cancer in patients younger than 40 years of age. J Surg Oncol.

[5] Nagashima O, Ohashi R, Yoshioka Y, Inagaki A, Tajima M, Koinuma Y, Iwakami S, Iwase A, Sasaki S, Tominaga S (2013). High prevalence of gene abnormalities in young patients with lung cancer. J Thorac Dis.

[6] VandenBussche CJ, Illei PB, Lin MT, Ettinger DS, Maleki Z (2014). Molecular alterations in non-small cell lung carcinomas of the young. Hum Pathol.

[7] Wang Y, Chen J, Ding W, Yan B, Gao Q, Zhou J (2015). Clinical features and gene mutations of lung Cancer patients 30 years of age or younger. PLoS One.

[8] Catania C, Botteri E, Barberis M, Conforti F, Toffalorio F, De Marinis F, Boselli S, Noberasco C, Delmonte A, Spitaleri G (2015). Molecular features and clinical outcome of lung malignancies in very young people. Future Oncol.

[9] Sacher AG, Dahlberg SE, Heng J, Mach S, Janne PA, Oxnard GR (2016). Association between younger age and targetable genomic alterations and prognosis in non-small-cell lung Cancer. JAMA Oncol.

[10] Tanaka K, Hida T, Oya Y, Yoshida T, Shimizu J, Mizuno T, Kuroda H, Sakakura N, Yoshimura K, Horio Y (2017). Unique prevalence of oncogenic genetic alterations in young patients with lung adenocarcinoma. Cancer.

[11] Luo Wenxin, Tian Panwen, Wang Yue, Xu Heng, Chen Lu, Tang Chao, Shu Yang, Zhang Shouyue, Wang Zhoufeng, Zhang Jun, Zhang Li, Jiang Lili, Liu Lunxu, Che Guowei, Guo Chenglin, Zhang Hong, Wang Jiali, Li Weimin (2018). Characteristics of genomic alterations of lung adenocarcinoma in young never-smokers. International Journal of Cancer.

[12] Ye T, Pan Y, Wang R, Hu H, Zhang Y, Li H, Wang L, Sun Y, Chen H (2014). Analysis of the molecular and clinicopathologic features of surgically resected lung adenocarcinoma in patients under 40 years old. J Thorac Dis.

[13] Kim L, Kim KH, Yoon YH, Ryu JS, Choi SJ, Park IS, Han JY, Kim JM, Chu YC (2012). Clinicopathologic and molecular characteristics of lung adenocarcinoma arising in young patients. J Korean Med Sci.

[14] Cancer IAfRo (2015). WHO Classification of Tumours of the Lung, Pleura, Thymus and Heart.

[15] Goldstraw P, Chansky K, Crowley J, Rami-Porta R, Asamura H, Eberhardt WE, Nicholson AG, Groome P, Mitchell A, Bolejack V (2016). The IASLC lung Cancer staging project: proposals for revision of the TNM stage groupings in the forthcoming (eighth) edition of the TNM classification for lung Cancer. J Thorac Oncol.

[16] Cai W, Li X, Su C, Fan L, Zheng L, Fei K, Zhou C, Manegold C, Schmid-Bindert G (2013). ROS1 fusions in Chinese patients with non-small-cell lung cancer. Ann Oncol.

[17] Weixiang Zhong JZ, Huang K, Zhang J, Chen Z (2018). Comparison of clinicopathological and molecular features between young and old patients with lung cancer. Int J Clin Exp Pathol.

[18] Etzel CJ, Amos CI, Spitz MR (2003). Risk for smoking-related cancer among relatives of lung cancer patients. Cancer Res.

[19] Pao W, Girard N (2011). New driver mutations in non-small-cell lung cancer. Lancet Oncol.

[20] Shi Y, Au JS, Thongprasert S, Srinivasan S, Tsai CM, Khoa MT, Heeroma K, Itoh Y, Cornelio G, Yang PC (2014). A prospective, molecular epidemiology study of EGFR mutations in Asian patients with advanced non-small-cell lung cancer of adenocarcinoma histology (PIONEER). J Thorac Oncol.

[21] Arcila ME, Chaft JE, Nafa K, Roy-Chowdhuri S, Lau C, Zaidinski M, Paik PK, Zakowski MF, Kris MG, Ladanyi M (2012). Prevalence, clinicopathologic associations, and molecular spectrum of ERBB2 (HER2) tyrosine kinase mutations in lung adenocarcinomas. Clin Cancer Res.

[22] Shigematsu H, Takahashi T, Nomura M, Majmudar K, Suzuki M, Lee H, Wistuba II, Fong KM, Toyooka S, Shimizu N (2005). Somatic mutations of the HER2 kinase domain in lung adenocarcinomas. Cancer Res.

[23] Inamura K, Takeuchi K, Togashi Y, Nomura K, Ninomiya H, Okui M, Satoh Y, Okumura S, Nakagawa K, Soda M (2008). EML4-ALK fusion is linked to histological characteristics in a subset of lung cancers. J Thorac Oncol.

[24] Takahashi T, Sonobe M, Kobayashi M, Yoshizawa A, Menju T, Nakayama E, Mino N, Iwakiri S, Sato K, Miyahara R (2010). Clinicopathologic features of non-small-cell lung cancer with EML4-ALK fusion gene. Ann Surg Oncol.

[25] Skaug V, Ryberg D, Kure EH, Arab MO, Stangeland L, Myking AO, Haugen A (2000). p53 mutations in defined structural and functional domains are related to poor clinical outcome in non-small cell lung cancer patients. Clin Cancer Res.

[26] Martelli MP, Sozzi G, Hernandez L, Pettirossi V, Navarro A, Conte D, Gasparini P, Perrone F, Modena P, Pastorino U (2009). EML4-ALK rearrangement in non-small cell lung cancer and non-tumor lung tissues. Am J Pathol.

[27] Vogelstein B, Lane D, Levine AJ (2000). Surfing the p53 network. Nature.

[28] Bergethon K, Shaw AT, Ou SH, Katayama R, Lovly CM, McDonald NT, Massion PP, Siwak-Tapp C, Gonzalez A, Fang R (2012). ROS1 rearrangements define a unique molecular class of lung cancers. J Clin Oncol.

[29] Auliac JB, Bayle S, Vergnenegre A, Le Caer H, Falchero L, Gervais R, Doubre H, Vinas F, Marin B, Chouaid C (2018). Patients with non-small-cell lung cancer harbouring a BRAF mutation: a multicentre study exploring clinical characteristics, management, and outcomes in a real-life setting: EXPLORE GFPC 02-14. Curr Oncol.

[30] Jing C, Mao X, Wang Z, Sun K, Ma R, Wu J, Cao H (2018). Nextgeneration sequencingbased detection of EGFR, KRAS, BRAF, NRAS, PIK3CA, Her2 and TP53 mutations in patients with nonsmall cell lung cancer. Mol Med Rep.

[31] Shigemitsu K, Sekido Y, Usami N, Mori S, Sato M, Horio Y, Hasegawa Y, Bader SA, Gazdar AF, Minna JD (2001). Genetic alteration of the beta-catenin gene (CTNNB1) in human lung cancer and malignant mesothelioma and identification of a new 3p21.3 homozygous deletion. Oncogene.

