# Supplementary material for: Molecular features of lung adenocarcinoma in young patients
Source: BMC Cancer. 2019 Aug 6;19:777. doi: 10.1186/s12885-019-5978-5 (PMC6685166; doi:10.1186/s12885-019-5978-5)
Supplement: Supplementary file 1 — Table S1. List of 59 cancer-associated genes. (DOC 35 kb) [file 12885_2019_5978_MOESM1_ESM.doc]

Table S1. List of 59 cancer-associated genes.

| *ABL1* | *AKT1* | *ALK* | *APC* | *ATM* | *BARF* | *CBL* |
| --- | --- | --- | --- | --- | --- | --- |
| *CDH1* | *CDK4* | *CDKN2A* | *CHEK2* | *CSF1R* | *CTNNB1* | *DNMT3A* |
| *EGFR* | *ERBB2* | *ERBB3* | *ERBB4* | *EZH2* | *FBXW7* | *FGFR1* |
| *FGFR2* | *FLT3* | *GNA11* | *GNAS* | *HNF1A* | *HRAS* | *IDH2* |
| *JAK1* | *JAK2* | *JAK3* | *KARS* | *KDR* | *KIT* | *MET* |
| *MLH1* | *MPL* | *NFE2L2* | *NOTCH1* | *NPM1* | *NRAS* | *PAX5* |
| *PDGFRA* | *PIK3CA* | *PPP2R1A* | *PTCH1* | *PTEN* | *RAF1* | *RB1* |
| *RET* | *SF3B1* | *SMAD4* | *SMARCB1* | *SMO* | *STAT3* | *STK11* |
| *TP53* | *U2AF1* | *VHL* |  |  |  |  |
